# Impact of HIV on HPV-related cancers in men who have sex with men: a review

**DOI:** 10.3389/fcimb.2024.1428491

**Published:** 2025-01-20

**Authors:** Zixuan Zhang, Yuying Xing, Tingdan Gong, Wanlin Li, Siwei Zhang, Lanlan Wei

**Affiliations:** ^1^ National Clinical Research Center for Infectious Diseases, The Third People’s Hospital of Shenzhen, The Second Hospital Affiliated to Southern University of Science and Technology, Shenzhen, China; ^2^ School of Medicine, Southern University of Science and Technology, Shenzhen, China

**Keywords:** human papillomavirus, human immunodeficiency virus, MSM, co-infection, cancer

## Abstract

Co-infection with human immunodeficiency virus (HIV) significantly increases the incidence of human papillomavirus (HPV) infection and HPV-related cancers among men who have sex with men (MSM). Conversely, HPV infection can also influence HIV acquisition rates. HIV-induced immune suppression may affect chromosomal stability, gene expression, protein function and other molecular components in MSM with HPV-related cancers. Additionally, HIV infection also alters cellular mechanisms by compromising immune responses and epithelial integrity. In this review, we reviewed the influence of HIV on specific HPV-related cancers in MSM, including oropharyngeal squamous cell carcinoma, penile cancer, and anal cancer. We integrated epidemiological data from the past five years and discussed diagnosis and treatment strategies. Overall, our review offers crucial insights into the underlying molecular and cellular mechanisms of these co-infection MSM patients. Our review aims to assist future research in developing effective treatment strategies for MSM with HIV/HPV co-infection.

## 1 Introduction

Human papillomavirus (HPV) is a widespread DNA virus with considerable public health consequences, accounting for nearly all cases of cervical cancer and a significant portion of other anogenital and oropharyngeal cancers (Ranjit et al., 2020). HPV has a prevalence of over 20% in men and accounts for 2% of male cancer cases (Ventimiglia et al., 2016). It is transmitted through sexual contact, skin-to-skin contact, and by infecting infants during delivery. Among them, sexual transmission is the most common way of transmission, primarily through vaginal and anal mucosa (Burchell et al., 2006), highlighting the significant cancer risk associated with men who have sex with men (MSM).

HPV can be classified into low-risk HPV (LR-HPV) and high-risk HPV (HR-HPV) according to its oncogenic potential (Handisurya et al., 2009). LR-HPV, including subtypes 6, 11, 42, and 43, is mainly associated with the formation of genital warts and may also cause warts in the oral cavity and throat area. HR-HPV subtypes, encompassing HPV 16, 18, 31, 33, 35, 39, 45, 51, 52, 56, 58, and 59, can lead to intraepithelial lesions and a range of HPV-related cancers, including cervical, oropharyngeal, penile, and anal cancers. Among these, HPV 16 and HPV 18 are the most carcinogenic (de Sanjose et al., 2018).

The HPV genome consists of three major regions. The long control region (LCR) is a non-coding region that regulates viral replication and transcription. The other two regions encode eight open reading frames (ORFs), including six early ORFs and two late ORFs. Early ORFs produce E1, E2, E4, E5, E6, and E7, which are involved in viral replication and tumorigenesis. The late ORFs are L1 and L2, which encode viral capsid proteins (Molina et al., 2024). HPV can invade the damaged epithelium, replicates extensively within it. Along with the amplification of HPV and cell division, resulting in a large number of cells becoming infected with HPV (Graham, 2017).

Persistent HR-HPV infection may lead to the development of cancer (Brickman and Palefsky, 2015). Due to the special physiological structure of the foreskin, men are more susceptible to HPV infection. In a study of 379 adult males, HPV infection rates varied by anatomical site and circumcision status. Uncircumcised men have higher rates of HPV in the glans or corona, as well as increased risk of oncogenic HPV and multiple HPV type infections (Hernandez et al., 2008). The excessive foreskin or phimosis, the inner foreskin and glans penis create a warm, humid, and anaerobic environment. This condition facilitates the proliferation of various anaerobic bacteria and viruses, including HPV (Mehta et al., 2021).

Human immunodeficiency virus (HIV) mainly invades CD4^+^T lymphocytes and destroys the host immune system, causing acquired immune deficiency syndrome (AIDS) (Deeks et al., 2015). HIV-1 and HIV-2 share similarities in genetic arrangement, transmission, replication, and clinical outcomes, both leading to AIDS. Although HIV-2 is less contagious and less likely to progress to AIDS, with its prevalence primarily confined to West Africa (Nyamweya et al., 2013), both HIV-1 and HIV-2 significantly influence HPV infection. Specifically, the incidence of HR-HPV is nearly doubled in HIV-positive individuals, with a pooled RR of 2.20 and a 95% confidence interval (CI) of 1.90 to 2.54, while the clearance rate of HPV is approximately halved, with a pooled RR of 0.53 (95% CI: 0.42 to 0.67) (Looker et al., 2018). HIV-positive MSM face a higher risk of HPV infections. An analysis enrolled 1,559 participants, including 300 HIV-positive MSM, 600 HIV-negative MSM, and 659 MSW (men who have sex with women), with HPV prevalence rates of 62.0%, 53.7%, and 8.3%, respectively (p < 0.001) (Bai et al., 2024).The incidence of HPV-related lesions and malignant cancers in individuals infected with HIV is significantly higher than in individuals not infected with HIV. A meta-analysis of 34 studies among Chinese MSM found high rates of multiple HPV infections in anogenital warts: 75.9% in HIV-positive and 41.7% in HIV-negative individuals (Zhou et al., 2021). A study identified 502 HPV-related cancers in HIV-positive Hispanics across 864,067 person-years. Excluding oropharyngeal cancer, the risk of HPV-related cancers was higher in HIV-infected patients than in the general population, with SIRs ranging from 3.59 for cervical cancer to 18.7 for anal cancer in men (Ortiz et al., 2018). HPV infection occurring before HIV can also impact HIV acquisition rates. A meta‐analytic systematic review of 14 publications showed HIV incidence was almost doubled (pooled RR = 1.91, 95% CI 1.38 to 2.65) in the presence of prevalent HPV infection (Looker et al., 2018). Understanding the bidirectional relationship between HIV and HPV is essential for effective prevention and treatment strategies for both infections.

**Figure 1:**
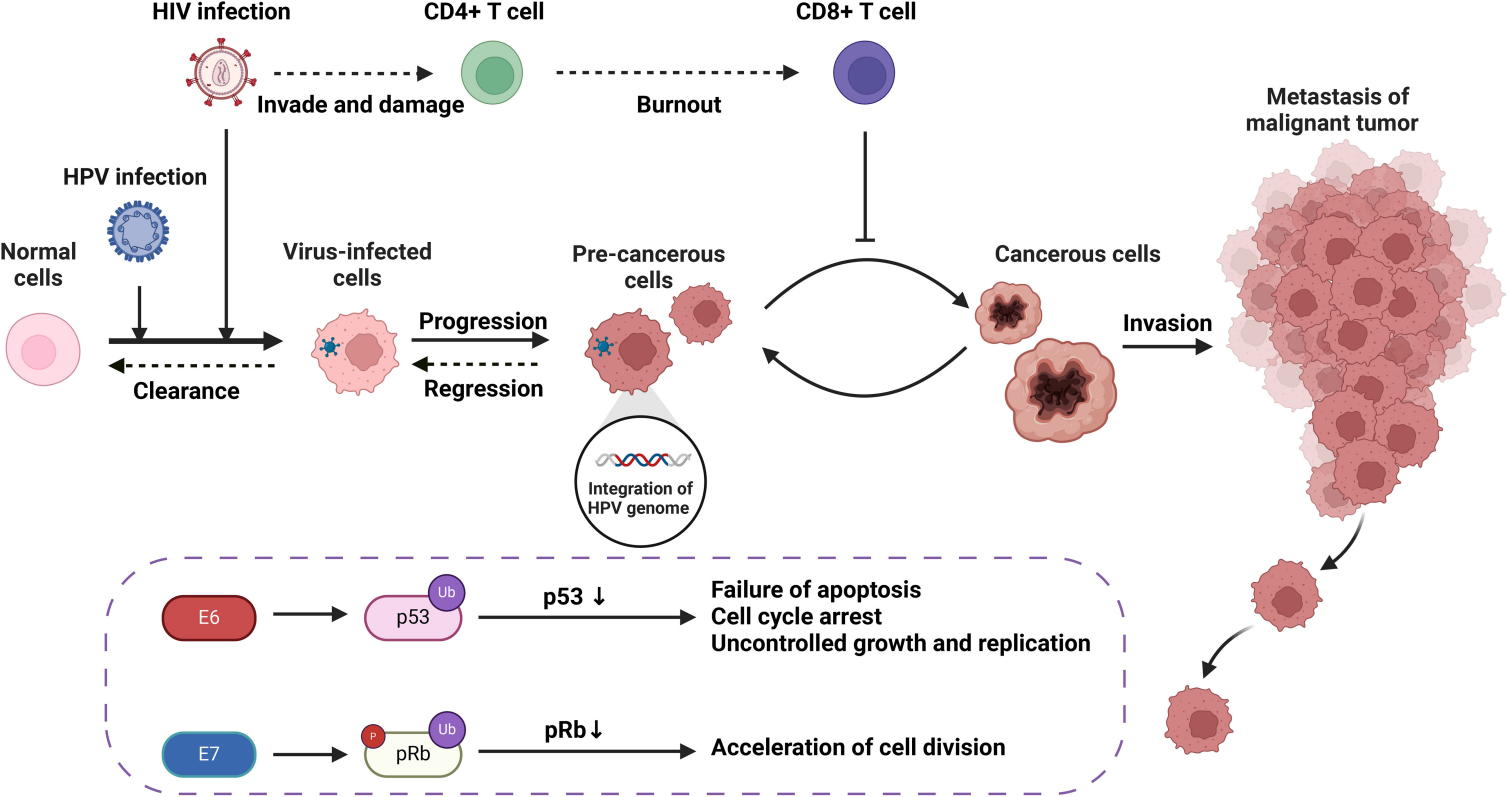
The carcinogenic progression of HPV-associated tumors resulting from HIV/HPV co-infection, including molecular and cellular mechanisms.

**Figure 2:**
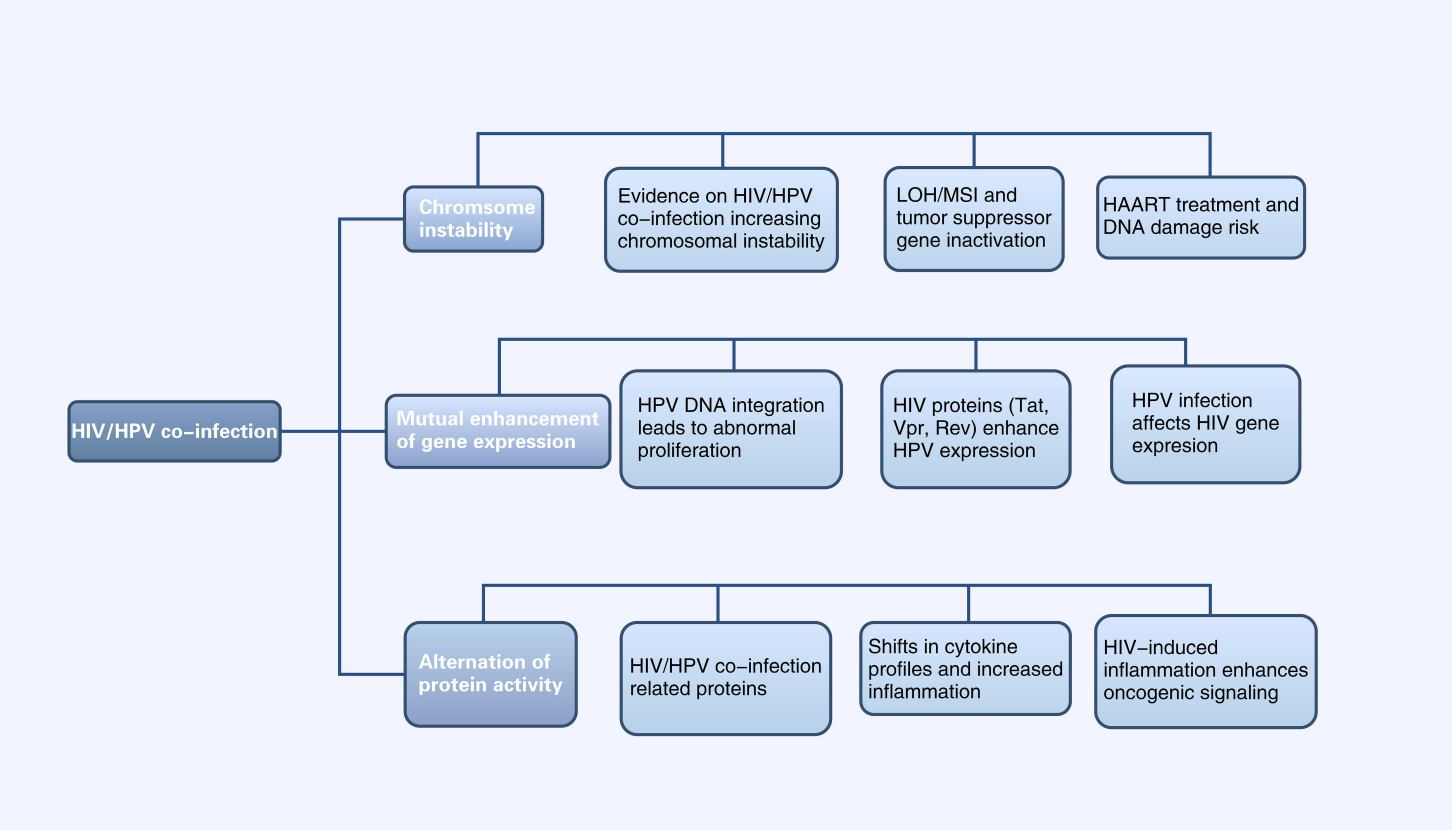
Diagram of Alterations at the molecular level of HPV-related cancers by HIV Infection (HIV infection can affect HPV-related tumor progression at the chromosomal, gene expression, and protein levels, and vice versa. LOH, Chromosomal heterozygous deletion; MSI, Microsatellite instability; HAART, Highly active antiretroviral therapy).

**Figure 3:**
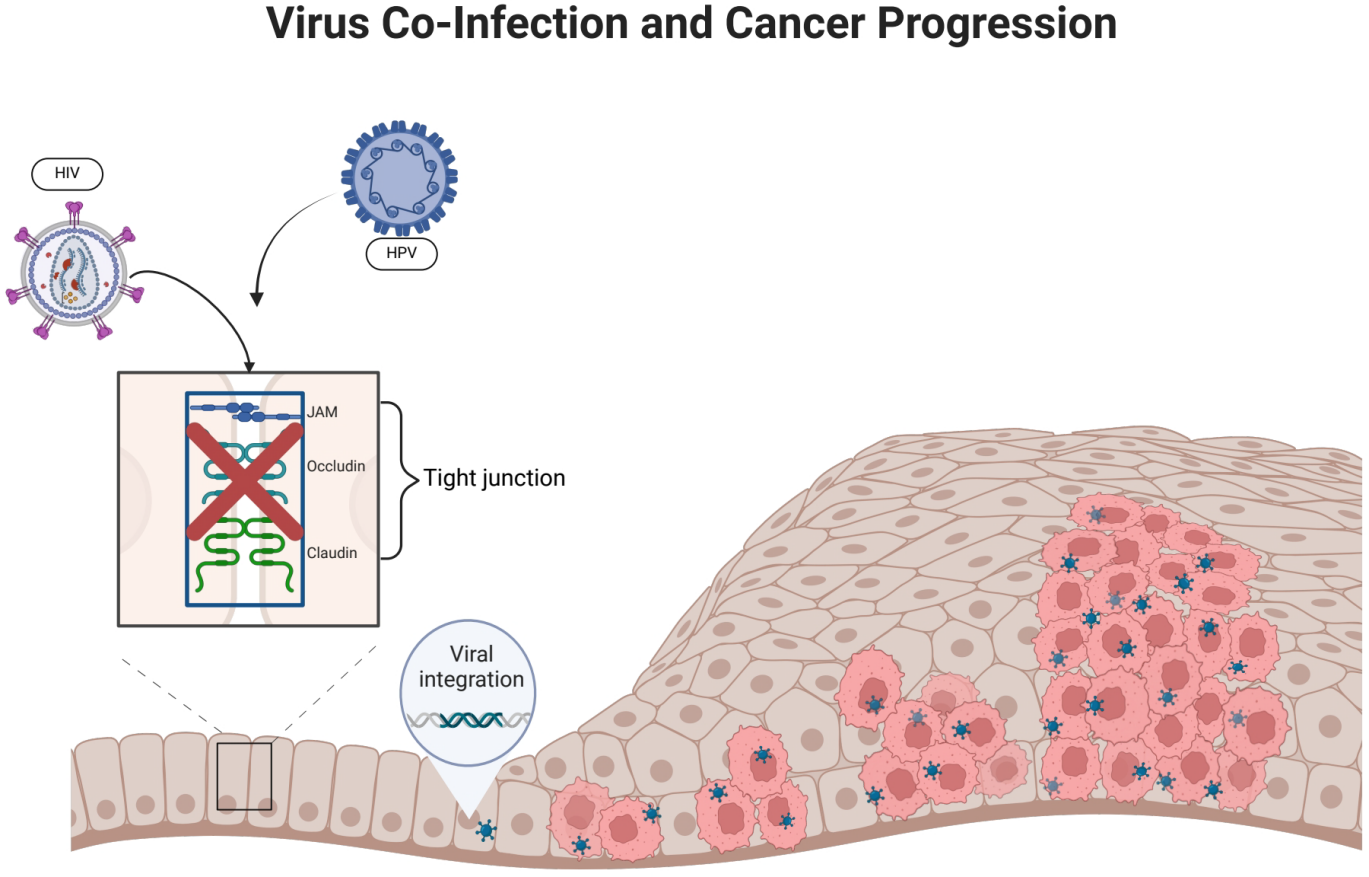
HIV infection promotes HPV infection. (HIV infection can disrupt epithelial tight junctions, thereby facilitating initial HPV infection. HPV enters epithelial basal cells with the help of surface proteins that bind to cellular receptors. The incorporation of HPV DNA into the host cell genome promotes abnormal cellular proliferation and differentiation).

**Figure 4:**
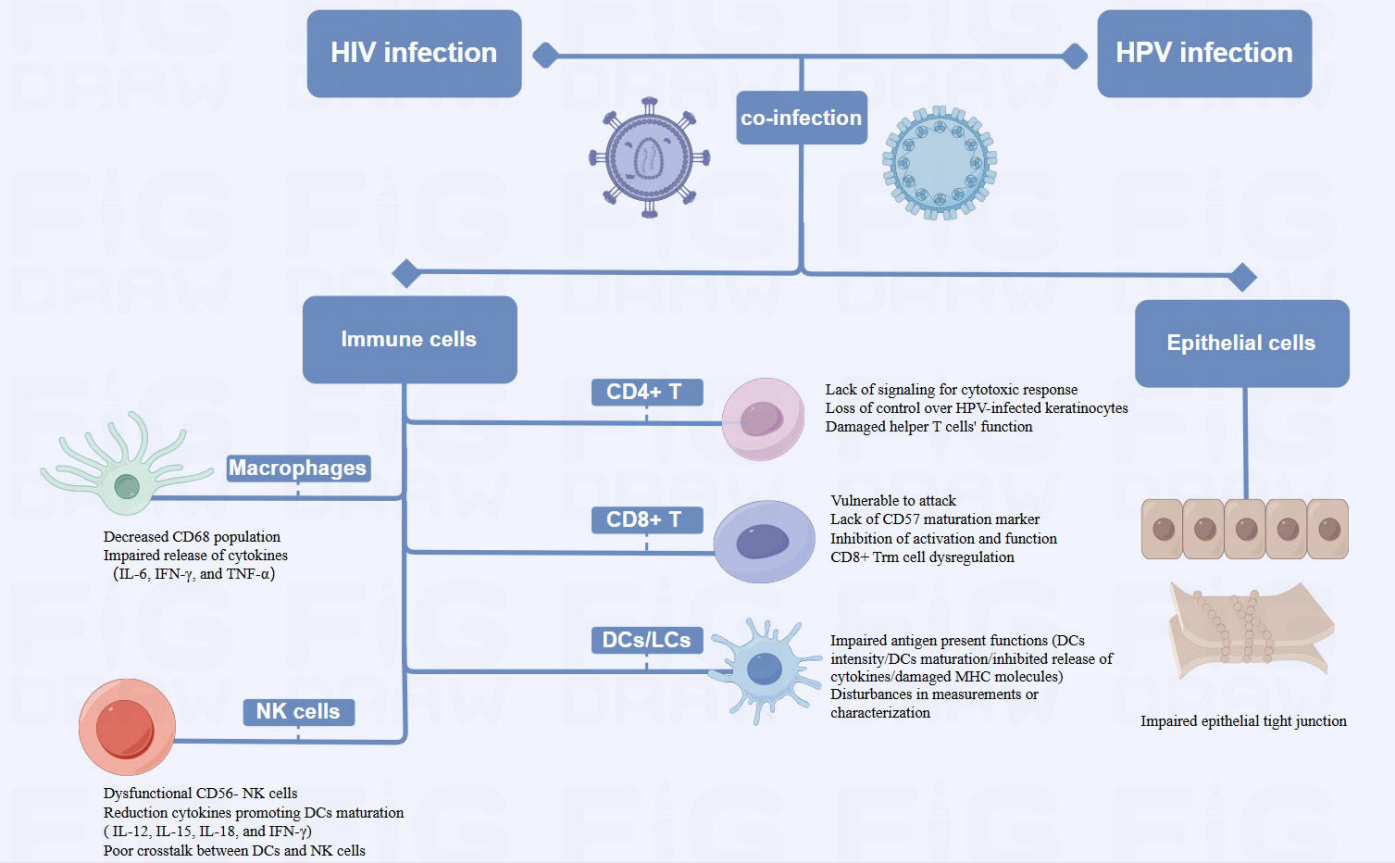
Diagram of Alterations at the cellular level of HPV-related cancers by HIV Infection (HIV-related immunosuppression, involving immune cells such as T cells, DCs/LCs, NK cells and Macrophages, can delay HPV clearance and increase cancer risk by reactivating or sustaining HPV infection. HIV damages epithelial tight junctions, facilitating HPV penetration and promoting HPV-related cancers).

In this review, we summarized the molecular mechanism of HIV affecting HPV carcinogenesis from chromosome, gene expression and protein levels (**Figures 1**, **2**). We further elaborate on the mechanisms of immune cell heterogeneity in the immunological microenvironment where HIV infection is prone to be complicated by HPV infection (**Figures 3**, **4**). Additionally, we collected epidemiological data from the past 3-5 years including anal cancer, penile cancer, and head and neck cancer. This data was scrutinized to identify the connections between HIV-positive MSM, HPV infection, and the associated cancers. We also categorized the diagnosis and treatment, and HPV vaccines for HPV-related cancers, offering insights for future prevention and intervention strategies.

## 2 Manuscript formatting

### 2.1 Alterations at the molecular level of HPV-related cancers by HIV Infection

#### 2.1.1 HIV/HPV co-infection enhances chromosomal instability

Loss of heterozygosity (LOH) of chromosomes and microsatellite instability (MSI) are important mechanisms leading to the inactivation of tumor suppressor genes (Edelmann et al., 2004). In cervical cancer, HPV can induce LOH/MSI at the DNA HLA-I locus, which is exacerbated when co-infected with HIV-1. A study revealed a significantly higher frequency of LOH/MSI at the HLA-I locus on chromosome 6p21.21 in HIV-1/HPV co-infected tumor DNA compared to HIV-1 seronegative patients (Chambuso et al., 2019). In addition, the integration of HPV genes in the host leads to chromosomal genetic changes, including chromatin reorganization and chromosome rearrangement (Li et al., 2019; Singh et al., 2024). The E6 and E7 proteins encoded by the HPV genome have been shown to lead to host chromosomal instability (Duensing and Münger, 2002). HIV infection can increase the expression of HPV E6 and E7 proteins (Tornesello et al., 2008), thus leading to host chromosomal instability. This mechanism will be further discussed in section 3.1.2. On the other hand, the current mainstay of treatment for HIV is highly active antiretroviral therapy (HAART), which includes some drugs, such as AZT, that have properties that may lead to DNA damage (Palefsky, 2006). Therefore, HIV/HPV-co-infected individuals treated with HAART may be at increased risk of chromosomal instability and, thus, more susceptible to cancer. In conclusion, HIV/HPV co-infection leads to chromosomal instability and increase the risk of HPV-related cancers.

#### 2.1.2 Interactions between HIV and HPV genes

The integration of HPV DNA into the host cell genome is a key event leading to abnormal proliferation and malignant progression in HPV-mediated carcinogenesis (McBride and Warburton, 2017). E6 and E7, two viral oncogenes that interfere with the pathways regulated by p53 and other members of the pRB family, are the primary cancer suppressor genes that cause neoplastic conversion (Chow, 2015). E6 inactivates p53, which is a transcription factor regulating the expression of genes encoding cell cycle, DNA repair mechanisms, metabolism, and apoptosis (Hafner et al., 2019). E7 inactivates pRb, keeping infected cells in a proliferative state. As a result, the continued activity of the E6 and E7 proteins leads to abnormal cell proliferation, the accumulation of oncogene mutations, and eventually HPV-related cancers (Faraji et al., 2017).

HIV proteins Tat, Vpr and Rev participate in HPV-related pathogenesis and increase the risk of HPV-associated cancers by affecting HPV gene expression (Nyagol et al., 2006). *Tat* encodes a transcription activator protein that can activate the HPV LCR and increase the expression of HPV E6, E7, E1, and L1 (Nyagol et al., 2006; Kim et al., 2008; Tornesello et al., 2008). Studies have shown that HIV-1 Tat1 protein can significantly upregulate HPV 16 gene expression by enhancing the activity of HPV 16 upstream regulatory region and its associated promoter (P97) (Vernon et al., 1993). Rev protein can increase HPV L1 gene expression by overcoming post-transcriptional inhibition. HPV-16 L1 gene expression is inhibited by specific sequences on mRNA. Rev protein can bind to the Rev-responsive element (RRE) on mRNA and effectively increase the expression of HPV L1 (Zheng and Baker, 2006). Vpr and E6 are linked in cell cycle signaling pathways. Cervical cancer cells are susceptible to HIV-1 vpr-induced cell cycle arrest, while coexpression of HPV-16 E6 exacerbates this effect (Toy et al., 2000). Currently there are no other studies on possible interactions between HPV and other HIV genes and the proteins they encode. While data are limited, studies suggested that the weakened immune response caused by HIV allows HPV-related diseases such as high-grade intraepithelial neoplasia to persist, providing time for genetic changes that lead to cancer progression (Palefsky, 2006).

HPV infection can also affect HIV gene expression: some studies reported that extracellular vesicles secreted by HPV-infected cervical cancer cells can enhance HIV-1 replication through oxidative stress pathways, suggesting that HPV infection could make cervical cancer cells more susceptible to HIV and facilitating a potential vicious cycle of synergistic expression between HIV and HPV (Ranjit et al., 2020).

#### 2.1.3 HIV infection affects protein activity in HPV infection pathways

p53 is a pro-apoptotic tumor suppressor factor, controlling cell proliferation, senescence, DNA repair, and cell death in cancer (Mao and Jiang, 2023). In cervical cancers induced by HR-HPV, p53 is degraded by HPV protein E6 (Martinez-Zapien et al., 2016). Current research indicates that extracellular HIV-1 Tat protein can be taken up by human cervical cancer cells, followed by an increase in the expression of HPV E6 protein and a decrease in the levels of the cellular suppressor protein p53 (Barillari et al., 2016). The impact of HIV on p53 may due to the activation of cellular pathways that are different from those in HIV-negative lesions; however, no studies currently exist, and more research and further validation is needed. HIV/HPV co-infection significantly increases VEGF and p27 expression (Nicol et al., 2008). VEGF serves as an early indicator of cervical cancer development (Branca et al., 2006), while p27, a cyclin-dependent kinase (CDK) inhibitor, is crucial for cancer prognosis in various cancer types (Tjalma et al., 2005). ADAR1, an adenosine deaminase, regulates RNA editing and its dysregulation may contribute to cancer. A genetic study in HPV/HIV co-infected individuals found ADAR1 variants linked to HPV relapse (Pujantell et al., 2019).

It is known that HPV-infected patients have increased expression of IL-6 and TNF-α, and HIV infection may further enhance the carcinogenicity of HPV through the expression of pro-inflammatory cytokines such as IL-6 and TNF-α (Nicol et al., 2005a). Another study found that HIV/HPV co-infection was more predictive of a predominance of type 2 cytokines (IL-4, IL-10 and IL-6) compared to HPV-monoinfection (Behbahani et al., 2007). This shift of cytokines weakens the immune response to HPV-related cancers. Type 1 cytokines (IL-2, IFN-γ) enhance cellular and humoral immunity, leading to better clinical outcomes in HPV-related cancers. In contrast, Type 2 cytokines (IL-4, IL-10, IL-6), more prominent locally or peripherally, are often associated with humoral immune responses and suppress cell-mediated immune responses, which may promote the development of cervical squamous intraepithelial neoplasia (SIL) and change cancers (Nicol et al., 2005a). Cyclooxygenase-2 (COX-2) is an enzyme that plays a crucial role in inflammation and pain by catalyzing the conversion of arachidonic acid into prostaglandins. HPV proteins E6 and E7 can trigger the transcription of COX-2 (Subbaramaiah and Dannenberg, 2007). In cervical intraepithelial neoplasia (CIN) and cervical cancer, COX-2 is overexpressed and associated with poor prognosis (Sales et al., 2001). The combination of HIV infection may increase the carcinogenic risk of HPV-related lesions and tumors by inducing COX-2 levels. Research shows patients with HIV/HPV co-infection and squamous intraepithelial neoplasia have higher levels of COX-2 in their cervical cells than those infected only with HIV (Fitzgerald et al., 2012).

In addition, HIV infection activates signaling pathways associated with epithelial-mesenchymal transition (EMT), which increases the proliferative and metastatic capabilities of epithelial cancer cells, thus promoting the development and metastasis of cancer in the context of HIV/HPV coinfection (Tugizov, 2016). Research demonstrated that exposure to HIV-1 gp120 and tat or cell-free virions in HPV-immortalized epithelial cells results in the disruption of epithelial junctions, which initiates the EMT. This transition is a significant contributor to tumorigenesis. The transforming growth factor-beta (TGF-β) signaling pathway serves as the principal canonical network governing EMT in cancer contexts, which is predominantly regulated by the transcription factor in response to mitogen-activated protein kinase (MAPK) signaling. Inhibition of the MAPK and TGF-β pathways has been found to prevent EMT triggered by HIV-1 in oral epithelial cells, potentially leading to new treatment methods for cancers linked to HIV/HPV coinfection (Tugizov, 2016).

### 2.2 Alterations at the cellular level of HPV-related cancers by HIV infection

#### 2.2.1 Immune cells

HIV-infected patients may reactivate or sustain an HPV infection due to immune system suppression, which delays HPV clearance and raises the probability of cancer (Frisch et al., 2000; Mooij et al., 2016). HIV-related immunosuppression primarily involves a variety of immune cells, CD4^+^ T cells, CD8^+^ T cells, dendritic cells (DCs), natural killer (NK) cells and macrophages.

HIV infection causes a decrease in CD4^+^ T cells, which subsequently reduces the amount of immune cells in the body and exacerbates immunosuppression (Petry et al., 1994). Low CD4^+^ cell counts impair the immune system’s ability to clear HR-HPV infections, due to the lack of effective signaling for a robust cytotoxic response and increase the incidence of HPV-related cancers (Chaturvedi et al., 2009; Hewavisenti et al., 2023). Consequently, HIV-infected individuals experience reduced host clearance of HPV and relatively undisturbed epithelial growth, contributing to the progression of HPV-related cancers (Frisch et al., 2000). Several studies have also shown that failure to develop an effective cell-mediated immune (CMI) response leaves HPV-positive individuals vulnerable to persistent infection and increases the probability of progression to invasive carcinoma. HPV-infected keratinocytes downregulate their innate immune signaling pathways, leading to a failure in the release of proinflammatory cytokine, particularly type I interferons. Moreover, there are insufficient or absent signals for the activation and migration of langerhans cells (LCs), as well as for the recruitment of macrophages and stromal dendritic cells (DCs) (Stanley, 2012). The loss of CMI response control over HPV-infected keratinocytes began when CD4^+^ levels were significantly higher than 200/μL (Frisch et al., 2000). Additionally, the helper T cell function may be compromised by the functional deficit with reduced expression of IL-2, IFN-γ, and IL-4 in HIV-infected patients (Nicol et al., 2005b; Dhasmana et al., 2008). Th2 phenotype may predominate in the immune response. This shift may result in an inadequate cellular immune response necessary for clearing HPV infections, making co-infected individuals more susceptible to persistent HPV (Mahnke et al., 2012). In addition to supporting tissue homeostasis and immune defense, resident memory T (Trm) cells exert antimicrobial and anticancer properties (Gebhardt et al., 2018). Studies have revealed that CXCR3^+^ CD4^+^ T cells were critical in preventing persistent HPV infection and cancer progression (Bodily and Laimins, 2011; Hickman et al., 2015), while CXCR3^+^ CD4^+^ Trm cells were irreversibly depleted in patients with advanced HIV disease (Saluzzo et al., 2021). The irreversibly depleted cells may impede HPV control, which in turn increased cancer risk. Although HIV may accelerate the growth of HPV-related cancers by reducing CD4^+^ T cells, this mechanism is still up for debate. Several studies have shown that high CD4^+^ T cell counts did not significantly reduce HPV incidence. Reactivation of a latent infection and recent sexual encounter were two possible causes of incident infections (Critchlow et al., 1998). Poor immune management of precancerous lesions may also encourage the development of cancer (Palefsky and Holly, 2003), while HIV-mediated immunosuppression may not always result in late progression to invasive cancer. The progression of anogenital carcinoma *in situ* may be depend on the accumulation of extra genetic damage which occurs more quickly in rapidly proliferating epithelial cells with defective cell cycle regulation (Critchlow et al., 1995). These findings emphasized the complex immunologic relationship between HIV infection and HPV-related cancer development.

The removal of HPV-infected epithelial cells and the regression of lesions caused by infection may be aided by T lymphocytes, especially cytotoxic T lymphocyte activation (Chihu-Amparan et al., 2023). HPV oncoproteins E6 and E7 are presented by major histocompatibility complex I (MHC-1), rendering infected cells vulnerable to assault by CD8^+^ T lymphocytes (Scott et al., 2001). HIV-induced chronic inflammation depletes CD8^+^ T cells by up-regulating PD-1 expression, weakening the anti-cancer response (Bushara et al., 2022). Cervical biopsies from HIV-positive women show an increase in CD8^+^ T cells, however, most of these cells are CD45RO^+^ and lack CD57 markers, which reduces their capacity for cytolysis (Olaitan et al., 1996). High levels of type 2 cytokines induced by HIV infection also diminish CD8^+^ T cell function (Kim et al., 2008). CD8^+^ Trm is highest in the epidermis, but patients with advanced HIV develop irreversible CD8 Trm cell dysregulation, which may be one of the important reasons for the cancer susceptibility environment (Saluzzo et al., 2021).

Dendritic cells, particularly langerhans cells capture antigens and stimulate T cells to initiate an immune response. HIV affects their ability to stimulate T cells by inhibiting the release of cytokines like IL-12, lowering DC density, attenuating DC maturation, and deducing MHC molecule expression (Pachiadakis et al., 2005; Guimarães et al., 2011). In addition, disturbances in the measurement and/or characterization of LCs could potentially impede the immunological monitoring of HPV-related cervical lesions (Hubert et al., 2001), hence contributing to the local and systemic immune responses to HPV-induced cancers (Wright-Browne et al., 1997; Hachisuga et al., 2001).

In HIV-positive individuals, NK cell function is impaired, with a shift towards dysfunctional CD56^-^ NK cells. The cells exhibit diminished cytotoxic capabilities and impaired cytokine production (Mavilio et al., 2005). This dysfunction correlates with the plasma HIV load (Bere et al., 2014), and weakens the immune system’s overall antiviral response, facilitating persistent HPV infections. Additionally, DCs functioning is impaired during acute HIV infection, producing fewer IL-12, 15 and -18, and lowers IFN-γ produced by NK cells, which retrospectively results in poor DC maturation. Poor crosstalk between DCs and NK cells, leads to weakened, non-specific and abnormal immunity, resulting in poor control of opportunistic infections like HPV (Hens et al., 2016).

Macrophages, like CD4^+^ T cells, serve as the primary target cells for HIV and facilitate in the virus’s dissemination throughout the body. HIV infection significantly alters the body’s immune response to HPV infection, as evidenced by a decrease in the local CD68 population, and by affecting the expression of IL-6, IFN-γ, and TNF-α by macrophages. These changes collectively contribute to the cancer progression (Nicol et al., 2005c).

#### 2.2.2 Epithelial cells

A stratified squamous epithelium covers the mucous membranes of the oral cavity, cervix, and genital tract. It forms tight junctions, maintains morphology and physiological function, and acts as a physical barrier against infection (Sufiawati and Tugizov, 2014). HIV can damage epithelial tight junctions by interacting with mucosal epithelial cells through its envelope proteins gp120 and Tat (Tugizov, 2016). Following this interaction, HPV reaches the basal cell layer, where the life cycle of HPV infection begins. This mechanism not only enhances the penetration of HPV pseudoviral particles into the epithelium and causes an initial HPV infection, but it also facilitates the entry of HPV into the mucosal epithelium and promotes the development of HPV-related cancers. It may have been due to inflammatory factors such as tumor necrosis factor-α and TGF-β, as well as calcium mucus proteins and tightly linked proteins, which have been adjusted to the upper membrane cells after HIV infection (Hewavisenti et al., 2023). In addition, it was discovered that in approximately 60% of HIV-infected patients, epithelial junctions in the oral and anal mucosa epithelial tissues were disrupted, thereby confirming the role of HIV in amplifying the oncogenic potential of HPV (Tugizov et al., 2013).

### 2.3 Epidemiology, diagnosis and treatment of HIV and HPV co-infection

#### 2.3.1 Oropharyngeal squamous cell carcinoma

HPV is thought to be responsible for approximately 70% of oropharyngeal squamous cell carcinoma (OPSCCs) (Brickman et al., 2019). According to the global statistics reported in 2021, the percentage of HPV-positive OPSCCs was 33%, with significant regional variations in prevalence, ranging from 0% to 85% (Carlander et al., 2021), and men were far more likely than women to develop HPV-positive OPSCCs due to sexual behavior and number of sexual partners (Lechner et al., 2022).Furthermore, HIV-positive MSM patient are 1.5 to 2 times more likely to be infected with HR-HPV types or even have OPSCCs than HIV-negative MSM. The global prevalence of oral/oropharyngeal HPV infection in men was about 5%. Additionally, the overall prevalence of oral HPV infection was found to be 17.3% (95% CI: 13.6%-21.7%) in 9619 MSM from 14 different countries in the world, with a larger prevalence range of 3.8%-81.9% (p < 0.01). Furthermore, HIV-positive MSM had a greater pooled prevalence of oral HPV than HIV-negative MSM (22.5% vs. 14.5%; p = 0.1) (Farahmand et al., 2021). HPV 16, the most common HPV genotype in OPSCCs (Lechner et al., 2022), and the prevalence of HPV 16 was higher among HIV-positive MSM (4.7%; 95%CI 2.1–7.3) vs. 3.0%; 95%CI 0.5–5.5) in HIV-negative MSM. Furthermore, the analysis of 26 publications focusing on MSM concluded that HIV-positive MSM patients were susceptible to all types of HPV (28.9% vs. 17.1%), especially HR-HPV (16.5% vs. 9.1%) (King et al., 2016; Rossotti et al., 2024).

According to the UK National Multidisciplinary Guidelines, clinical examination includes direct flexible endoscopy of the upper aerodigestive tract and imageological examination, especially PET-CT and MRI. The former is used to assess the size and respectability of the primary tumor, while the latter is employed to assess the extent of lymph node disease and bone invasion and to detect distant metastases of the lung and liver (Mehanna et al., 2016). Confirmation of the diagnosis requires routine histopathologic examination, and immunohistochemistry may be added if necessary. PCR, DNA *in situ* hybridization and other methods can be utilized to evaluate the HPV status (Umudum et al., 2005; Bishop et al., 2012; Holzinger et al., 2012; Guo et al., 2014; Johnson et al., 2020). The combination of immunohistochemical staining of p16 and *in situ* hybridization of HR-HPV showed acceptable levels of sensitivity (97%) and specificity (94%) and could be performed using formalin fixed paraffin-embedded tissue (Lechner et al., 2022). Positive cases can also be identified by immunocytochemical p16^INK4a^/Ki67 double staining (Linxweiler et al., 2015). Treatment options for HPV-related OPSCC include surgery, radiotherapy, and chemoradiotherapy (CRT) (Lee et al., 2018; Gillison et al., 2019; Mehanna et al., 2019). Surgery includes open surgery and minimally invasive surgery, the latter mainly including transoral laser microsurgery and transoral robotic surgery (Lechner et al., 2022). Early (T1-T2, N0) OPSCCs can be treated with primary surgery or radiation therapy alone. Locally advanced (T3-T4, N0 and T1-T4, N1-N3) OPSCCs require multimodal treatment, including pre-operative surgery followed by RT or CRT, or eventual CRT, depending on pathological findings (surgical margin, extratodular extension) (Bozec et al., 2021). Three-weekly (100 mg/m²) cisplatin-based concurrent CRT should be the standard of care for patients with locally advanced HPV-related OPSCC (Gillison et al., 2019; Mehanna et al., 2019). Additionally, immunotherapy (Vermorken et al., 2008; Fakhry et al., 2014; Ferris et al., 2016) and HPV vaccination (Berenson et al., 2022) are also therapeutic and preventive tools. In two prospective studies, the anti-EGFR monoclonal antibody cetuximab has been investigated as an alternative to cisplatin to reduce the risk of treatment-related toxicity and morbidity. The anti-PD-1 antibodies nivolumab and pembrolizumab were first approved by the FDA in 2016 for patients with metastatic platinum-resistant OPSCC. Several therapeutic vaccines targeting the E6 and/or E7 have entered clinical trials in HPV OPSCC patients (Lechner et al., 2022). The prognosis for HPV-positive OPSCC is good, but 10-25% of patients will relapse. The National Comprehensive Cancer Network recommends screening every 1 to 3 months during the first year, every 2 to 6 months in the second year, every 4 to 8 months until the fifth year, and then annually thereafter. HPV DNA has proven to be a useful biomarker for monitoring disease status after treatment (Ellis et al., 2021).

#### 2.3.2 Penile cancer

Based on the statistics, the incidence of penile cancer worldwide was expected to be 0.8/100,000 in 2020, with Asia accounting for 56.3% of total cases (Sung et al., 2021). Furthermore, HPV is responsible for more than 75% of penile intraepithelial neoplasia and over 50% of penile cancers. According to the results of a recent meta-analysis, the overall pooled prevalence of penile HPV infection in MSM was 36.2% (95% CI:29.1%-44.0%), and the most frequent HR-HPV types in the penis were HPV 16 (4.9%, 95% CI: 3.6%–6.7%) and HPV 18 (3.2%, 95% CI: 2.4%–4.0%). Furthermore, HIV status increases the risk of penile HPV infection. According to the statistics, the pooled prevalence of penile HPV was substantially higher in HIV-positive MSM than it was in HIV-negative MSM (45.4%, 95% CI: 35.2%–56.0% vs. 28.6%, 95% CI: 19.4%–39.9%, respectively; p = 0.02) (Farahmand et al., 2020). HIV infection, as an independent risk factor, had a higher risk of penile cancer (RR = 3.7-5.8, three studies; SIR = 3.8-11.1, four studies) and had a four-fold increased risk of death. Additionally, progression from intra-epithelial neoplasia to cancer occurs 6 years earlier in HIV-positive men compared to HIV-negative men (Amini et al., 2023).

Penile cancer can be diagnosed by detecting HPV DNA using PCR, hybrid capture (HC) and 5% acetic acid. The first two methods demonstrate good sensitivity and correlation, while penoscopy has good specificity but low sensitivity (Figliuolo et al., 2012). The key to confirming the diagnosis is biopsy, and prognostic factors such as tissue subtype, grading, and cancer staging affect the outcome. Lymph node evaluation has important prognostic value and is closely related to the selection of adjuvant chemotherapy (Hakenberg et al., 2018). Treatment of penile cancer includes surgery, radiotherapy, chemotherapy, and immunotherapy. Local immunotherapy, chemotherapy, and laser ablation for *in situ* penile cancer (Manjunath et al., 2017). For patients with locally advanced penile cancer, and for those with fixed inguinal lymph nodes confirmed by biopsy, large >4 cm, bilateral and/or positive pelvic lymph nodes, neoadjuvant chemotherapy (NAC) is recommended, followed by surgical lymph node treatment, including inguinal lymph node dissection (ILND)/pelvic lymph node dissection (PLND). The preferred NAC standard schemes are four cycles of paclitaxel, ifosfamide, and cisplatin. These have been recommended by the National Comprehensive Cancer Network (NCCN) and European Association of Urology (EAU) guidelines (Chadha et al., 2022). In the case of locally advanced cancer, current NCCN guidelines recommend adjuvant chemotherapy in the form of TIP or 5-fluorouracil (5-FU) for patients who do not receive first-line NAC and exhibit ≥2 positive lymph nodes or extratodular expansion at ILND. For penile cancer patients with distant metastatic disease, the NCCN recommends chemotherapy as first-line treatment, using either the TIP or 5-FU plus cisplatin, followed by surgery or salvage systemic therapy for responders (Joshi et al., 2022a). Immunotherapy includes immune checkpoint blockade (ICB), HPV vaccines, adoptive T-cell therapies and engineered T-cell therapies, as well as tyrosine kinase inhibitors and other targeted therapies (Joshi et al., 2022b). In penile cancer, ICB is performed with anti-PD-L1, anti-PD-1, or anti-CTLA4 drugs. However, ICB approval is limited to second-line therapy for patients with relapsed or metastatic disease. Adoptive T-cell therapies to enhance T-cell-mediated tumor destruction, such as tumor infiltrating lymphocytes (TIL) therapy, chimeric antigen receptor T cell (CAR-T) therapy and engineered T cell receptor T cell (TCR) therapy. At present, the application of these therapies in the treatment of penile cancer remains in the research and exploratory stages. Current Phase I trials demonstrate the safety and efficacy of HPV targeting E6 or E7 cancer proteins. In terms of targeted therapy, anti-EGFR, and pan-HER TKI agents may be viable options for patients who are not candidates for standard of care combination chemotherapy or for those who have undergone platinum chemotherapy (Chadha et al., 2022).

#### 2.3.3 Anal cancer

Anal cancer accounts for approximately 2% of all intestinal mucosal malignant tumors. HPV infection and immunosuppression are the main risk factors for anal cancer.HPV-16 accounts for 85% of total squamous cell carcinoma of the anus (SCCA) (Muresu et al., 2020). The incidence of SCCA has increased annually over the past few decades by 2% to 6%, according to studies from high- and middle-income nations (Deshmukh et al., 2023). Anal cancer is rare in the general population, but its incidence has increased significantly among people living with HIV. A ten-year retrospective study conducted in Rome showed that HIV-positive men were about 1.4 times more likely to be HPV positive than HIV-negative men (Fracella et al). And Anal cancer is 19 times more common in HIV-positive patients than in the general population (Eng et al., 2022). HIV infection reduces the body’s clearance of HPV. HPV-16 clearance is about 1.6 times higher in HIV-negative MSM compared to HIV-positive MSM (Wei et al., 2023).

Diagnosing anal cancer involves history-taking, clinical recognition, biopsy, and MRI/CT. Recommended tests include HIV, p16, and HPV testing, along with PET-CT scans (Glynne-Jones et al., 2010). Screening methods for HIV patients include fingerprinting, anal Pap smear, HPV genotyping, and high-resolution anoscopy (Santorelli et al., 2018). Standard therapy involves chemoradiotherapy based on mitomycin C/cisplatin and 5-fluorouracil (Glynne-Jones et al., 2010). However, current treatments for anal cancer still have limitations. standard anal cancer treatments, especially in HIV-positive patients with low CD4 counts, require adjustments due to their toxicity and efficacy (Kauh et al., 2005). Many new treatment methods, such as targeted therapy, vaccination, immunotherapy, and photodynamic therapy, are undergoing clinical trials for the treatment of anal cancer (AC), and promising results have been achieved in certain indications.

#### 2.3.4 HPV vaccine

HPV vaccines, including bivalent (HPV2), quadrivalent (qHPV), and nine-valent (9vHPV) offer crucial protection against HPV infection. While HPV2 targets HPV 16 and 18, qHPV extends coverage to include HPV 6, 11, 16, and 18. The latest vaccine, 9vHPV, provides protection against five additional HR-HPV types: 31, 33, 45, 52, and 58.

The Advisory Committee on Immunization Practices (ACIP) has provided corresponding HPV vaccination recommendations for different age groups and genders. MSM and individuals with compromised immune systems, including those infected with HIV, should receive the vaccine up to the age of 26 (Markowitz et al., 2014). The Merck Manual emphasizes immunocompromised individuals, including those with HIV infection, can receive the three-dose series regardless of their age at the time of the initial vaccination (Savoy, 2024).

Clinical trials have demonstrated that the quadrivalent HPV vaccine shows good efficacy in males aged 16 to 26. In the early vaccination group, the incidence of various HPV-related diseases was significantly reduced, with an incidence rate of 0.0 per 10,000 person-years for HPV-6 or 11-related external genital warts, which is lower than the control group’s rate of 137.3. The incidence of external genital lesions related to HPV-6, 11, 16, or 18 types were also significantly reduced, with an incidence rate of 0.0 per 10,000 person-years. In high-risk MSM, the incidence of anal intraepithelial neoplasia or anal cancer caused by HPV-6, 11, 16, or 18 types decreased from 906.2 to 20.5 cases per 10,000 person-years (Goldstone et al., 2022).

Vaccination-induced antibody levels are comparable in HIV-infected patients to the general population, with HIV patients who have preserved CD4^+^ T cell counts showing higher HPV antibody titers (Mugo et al., 2018). Furthermore, the safety of HPV vaccination is better in HIV-positive MSM, with studies finding that the highest dose of therapeutic HPV type 16 vaccine administered was demonstrated to be safe and immunogenic (Gosens et al., 2023). Adding the qHPV vaccine to a regimen for the treatment of high-grade anal intraepithelial neoplasia (HGAIN), particularly for HIV-positive MSM aged ≥27 years, significantly reduces life-cycle costs and increases quality-adjusted life years (Deshmukh et al., 2015). In addition, dual vaccination targeted at both HPV and HIV proved to be a highly cost-effective strategy and being more cost-effective compared to any other pre-exposure (PrEP) strategy (Moodley et al., 2016). These findings provide helpful scientific support for the feasibility, efficacy, and application of HPV vaccines in the context of HIV-positive patients.

### 2.4 Summary and outlook

The impact of HIV on HPV-related cancers in males has been comprehensively examined in this study. This study elucidates molecular interactions, cellular regulatory mechanisms, and epidemiological correlations between HIV and HPV, underscoring their synergistic roles in cancer progression. It offers insights for precision therapies and comprehensive control strategies, including HPV vaccine efficacy assessments for future prevention and treatment.

However, several questions remain to be resolved in the future. Firstly, research on the interactions between HIV-2 and HPV and the impact of this co-infection on cancer development is minimal. Additionally, the long-term effects of HAART therapy on chromosomal stability in individuals co-infected with HIV/HPV need further exploration. The molecular pathways by which other HIV proteins are involved in HPV infection and the development of related cancers have not been fully elucidated, and the potential roles of other HIV proteins in the formation of HPV-related cancers also require investigation. Finally, while HPV vaccines have shown promising efficacy and safety in HIV-infected individuals, more long-term studies are needed to confirm these findings and optimize vaccination strategies, especially the potential benefits of dual vaccination targeting both HPV and HIV.
